# Poly(ethylene glycol) nanocomposites of sub-nanometer metal oxide clusters for dynamic semi-solid proton conductive electrolytes†
†Electronic supplementary information (ESI) available: Details of the *d-cals* calculation method, IR and impedance spectra, flow curves, relationship between the shear rate and stress, viscosities and volume fraction of POMs, cyclic curves of shear rate and shear stress, *E*_a_ of the nanocomposites, DLS data and the fitting curves of flow curves. See DOI: 10.1039/c9sc02779c


**DOI:** 10.1039/c9sc02779c

**Published:** 2019-07-11

**Authors:** Zhao Zheng, Qianjie Zhou, Mu Li, Panchao Yin

**Affiliations:** a South China Advanced Institute for Soft Matter Science and Technology , State Key Laboratory of Luminescent Materials and Devices , South China University of Technology , Guangzhou , 510640 , P. R. China . Email: yinpc@scut.edu.cn

## Abstract

Sub-nm-scale metal oxide clusters (PW_12_O_40_^3–^) show high solubility in the melt of poly(ethylene glycol) (PEG) and the obtained semi-solid nanocomposites show promising proton conductivities under ambient conditions. Suggested from scattering studies, the clusters are homogeneously dispersed in the PEG melt at the molecular scale with high loading amounts (70 wt%) and the formed real solutions can be stable for months with no aggregation or phase separation. The conductivities of the nanocomposites which are governed by the concentrations of H_3_PW_12_O_40_ can reach as high as 1.01 × 10^–2^ S cm^–1^ at the highest concentration. Due to the dynamic cross-linking hydrogen bonding between clusters and PEG, the nanocomposites behave like solids with negligible flow at high concentrations of clusters. Upon the application of high-speed shear forces (>32 s^–1^), the composites can flow with continuously decreasing viscosities. The shear thinning properties of the nanocomposites enable their convenient processing into required morphologies and the wettability of electrolytes to electrodes under typical high shear rate processing conditions and the safety of the produced devices can be ensured by their solid-like properties in the static state.

## Abstract 2

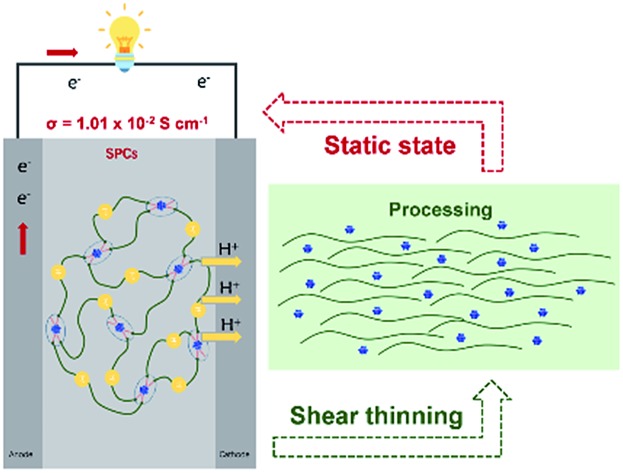
PEG–POM nanocomposites are ideal candidates for semi-solid proton conductors with high proton conductivities and devisable mechanical performances.

## Introduction

With energy and environmental issues becoming more prominent, proton conducting materials have received increasing interest in recent years for their broad applications in energy conversion and storage devices, including supercapacitors and fuel cells.^1^–^4^ To resolve the issue of leaking of electrolytes, solid and semi-solid proton conductors (SPCs) have been employed as electrolytes in capacitors and batteries, which can significantly decrease the risk for device failure and related safety issues.^5^–^9^ Owing to their appreciable mechanical properties, SPCs have also been under intense development for their applications as the key components in fuel cell devices: proton exchange membranes and proton conducting electrolytes.^1^,^10^–^12^ Meanwhile, the growing needs for portable and flexible power supplies have increased the demand for SPCs with low weight, high toughness and deformability, and superior processability.^13^–^17^ Crosslinked polyelectrolytes have been dominantly used for the manufacture of proton exchange membranes and electrolytes in fuel cells and supercapacitors.^4^,^11^,^16^,^18^ Further introduction of inorganic nano-materials has been used to enhance the thermal stabilities and conductivities of electrolytes.^19^–^21^ However, poor compatibility between the two components could lead to phase separation and the resulting unstable device performance.^22^ The study of the chemistry regarding the interaction between inorganic and polymer components is key to resolve the above issues.^22^ Meanwhile, the high elasticity of SPCs is of high importance to the stability and flexibility of devices, which, however, could significantly increase the difficulty and cost in materials processing. Moreover, highly relying on their flowability and deformability, the wettability and compatibility of electrolytes to electrodes are key to the performance of energy storage devices.^23^–^26^ Therefore, to develop strategies that can balance the mechanical performance, wettability to electrodes, and processability is critical in developing next generation commercializable SPCs.

Polyoxometalates (POMs) are a group of mono-disperse, nano-scale metal oxide clusters with well-defined structures and tunable electronic and surface properties.^27^–^32^ As the transition zone between small molecules and nanoparticles (NPs), sub-nm POMs (mainly Lindqvist and Keggin-types) have extremely high specific surface areas, admirable catalytic efficiencies and proton conductivities, and excellent stabilities.^33^–^39^ Especially, with poly(ethylene glycol) (PEG) molecules encapsulated inside the nano-channels defined by Keggin-type POMs, the obtained PEG@POM hybrid materials show comparatively high proton conductivities at high temperature (up to 443 K) and low humidity (<1% RH).^40^ It is reported in our recent work that proton conduction in the PEG@POM hybrids is basically facilitated by the diffusive motion of PEG chains and therefore the introduction of water into this system is not necessary.^41^ However, the dynamics of the PEG chain is severely slowed down by the strong confinement of PEG in the POM framework, limiting the proton conductivities of PEG@POM.^40^,^42^ The high crystalline energy inherited from POMs defines the poor mechanical properties and processability of PEG@POM. The structural optimization of PEG–POM hybrids is urgently required to resolve the above two issues. Meanwhile, PEG has been reported as a good solvent for POMs as media for catalytic reactions although the micro-structures and thermodynamic stabilities of such dispersion systems have not yet been studied.^33^,^43^ Due to the enriched oxo ligands on the POM's surface, hydrogen bonding (HB) interactions between POM and PEG have been confirmed in organic solvents by neutron scattering techniques.^44^ Therefore, to break the crystalline framework of POMs and free PEG out of the resulting nano-confinement, the volume fraction of PEG is significantly increased and POMs are expected to disperse homogeneously as single clusters in the PEG melt. In the as-obtained POM–PEG hybrids, protons are expected to be transported along the PEG chains and their proton conductivities can be optimized by altering PEG networks in the hybrids. Moreover, the HB among POMs and PEGs will crosslink the hybrids to SPCs with thermo- and shear-responsive properties resulting from the dynamic nature of HB. This smart behavior would provide us the capability to tune the flow properties, *e.g.* viscosity, to meet the requirements from the perspective of device processing, performance, and safety. Herein, as a model system, phosphotungstic acid (H_3_PW_12_O_40_, PW_12_), a typical commercially available robust Keggin type POM with a size of *ca.* 1 nm, is dispersed in PEG at different concentrations (10–70 wt%).^45^ The microstructures in the bulk of PEG–POM hybrids have been, for the first time, revealed by scattering techniques. The proton conductivities and dynamic mechanical properties of the hybrids have been optimized to the scales close to commercial requirements.^46^ Further thermodynamic studies are conducted for the exploration of the structure–property relationship of the hybrid nanocomposites.

## Experimental

### Materials and synthesis

PEG with a weight average molecular weight (*M*_w_) of 400 g mol^–1^ and H_3_PW_12_O_40_·*n*H_2_O were purchased and used directly without further purification. The synthetic protocol for PEG400–PW_12_ nanocomposites is as follows: crystalline samples of PW_12_ were added to PEG-400 at 340 K with vigorous stirring. The solutions were kept stirring at 340 K for 12 h, and then cooled to room temperature. The samples were stored in a vacuum oven at room temperature. The recipes for all the prepared samples used in this article are listed in the ESI (Table S1†).

### Measurements

Prior to all measurements, the samples were dried in a vacuum oven at room temperature for two weeks to remove water. Small angle X-ray scattering (SAXS) data were collected at the beamline BL16B1 of the Shanghai Synchrotron Radiation Facility (SSRF) with a Pilatus 2M detector. FT-IR spectra were measured in the range of 600–3600 cm^–1^ through the attenuated total reflectance (ATR) mode of a Nicolet iS5N. Viscoelastic properties of the PEG400–PW_12_ nanocomposites were studied using an Anton Paar Rheometer 302 (MCR302) equipped with a 25 mm diameter cone plate at 298 K, 323 K, and 353 K respectively. The shear rate range of zero shear viscosity measurements is 1–200 s^–1^. Alternating current (AC) impedance measurements of nanocomposites were performed using a CHI660E electrochemical workstation using two parallel platinum plate electrodes (area, *A* = 1 cm^2^, distance (*L*) between the plates is 1 cm) over 0.01 Hz to 100 000 Hz at 298 K, 323 K, and 353 K, respectively (RH = 45%). Proton conductivity was calculated using the formula *σ* = *L*/(*AR*_b_), and the electrolyte resistance (*R*_b_) was obtained from the intercept of the Nyquist plot with the real axis. DLS data were obtained by the dynamic light scattering method conducted on a Brookhaven BI-200SM research goniometer and laser light scattering system.

## Results and discussion

### Nanocomposite structures and solution stability

**Fig. 1:**
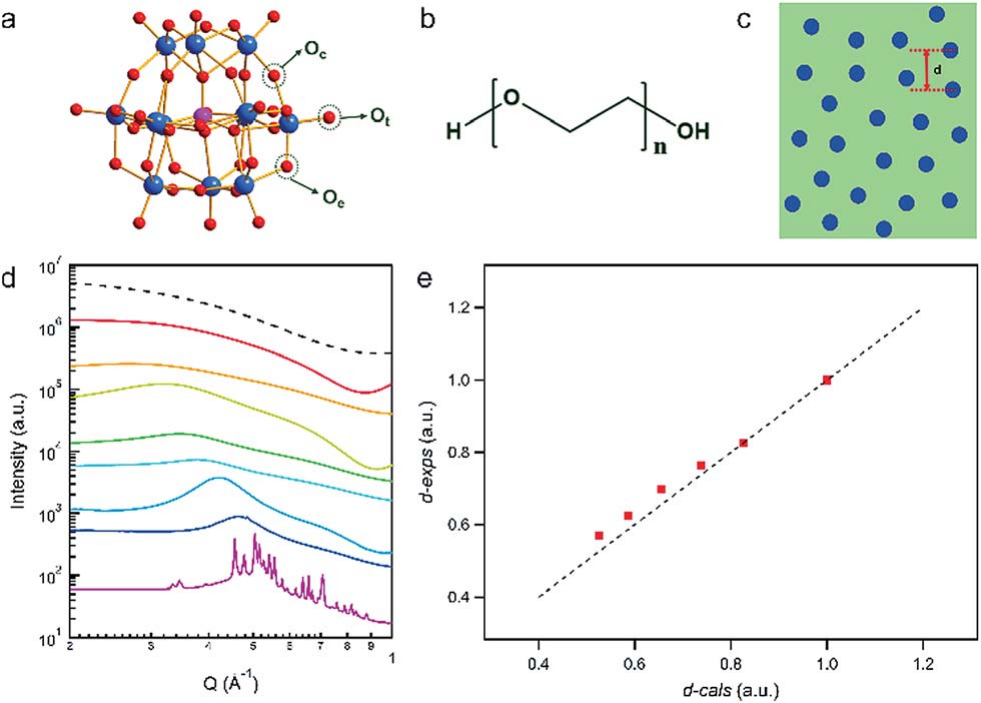
(a) Ball-stick model of PW_12_. (b) Molecular formula for PEG400. (c) Schematic of the inter-cluster distance. (d) SAXS curves of the PEG400–PW_12_ nanocomposites with different PW_12_ concentrations (black dashed line for the form factor of PW_12_ simulated from the single crystal structure of PW_12_, red for PEG400–10% PW_12_, orange for PEG400–20% PW_12_, yellow for PEG400–30% PW_12_, green for PEG400–40% PW_12_, cyan for PEG400–50% PW_12_, light blue for PEG400–60% PW_12_, dark blue for PEG400–70% PW_12_, and purple for PEG400–80% PW_12_). (e) Relationship between *d-exps* and *d-cals* (red square for raw data and dashed line represents the fitted curve *y* = *x*).

Upon gentle heating (∼340 K), the crystals of PW_12_ can be fully dissolved in the melt of PEG with a high concentration (*ca.* 70 wt%) and form clear, stable solutions when cooled to room temperature (Fig. 1a–c). To confirm the structures and stabilities of the nanocomposites, SAXS is applied to probe the solutions of PW_12_ clusters in PEG at different concentrations. Due to the high sensitivity of X-ray to heavy metal ions, SAXS is capable of collecting the form factor (*P*(*Q*)) of PW_12_ regarding its morphology, structure factor (*S*(*Q*)) information on the interaction among PW_12_ clusters and possible aggregation of PW_12_.^47^,^48^ For diluted solutions of PW_12_ (<20 wt%) in PEG, the measured *P*(*Q*) of PW_12_ in SAXS data is identical to that calculated from the PW_12_ molecular model, indicating that the molecular structure of the PW_12_ cluster is intact during the dissolving process (Fig. 1d).^49^ Moreover, only the *P*(*Q*) of PW_12_ can be observed in the SAXS data without the contribution of diffraction peaks of PW_12_ crystals and *S*(*Q*), suggesting that PW_12_ prefers to stay as discrete clusters in the PEG melt (Fig. 1d). When the concentrations of PW_12_ are comparatively higher (20–70 wt%), the interaction among PW_12_ clusters becomes strong and featured bumps, corresponding to *S*(*Q*), can be observed, which, typically, can be interpreted as the averaged inter-cluster distances (Fig. 1c).^50^ As the cluster concentrations become higher, the *S*(*Q*) peak positions shift to a high *Q* direction gradually, suggesting closer inter-cluster distances (Fig. 1d, from 2.38 nm at 20 wt% to 1.35 nm at 70 wt%). As a quantitative analysis, the inter-cluster center-to-center distances obtained from *S*(*Q*) in SAXS data (*d-exps*) are plotted against the theoretical inter-cluster distances derived from solution concentrations (*d-cals*) (Fig. 1e) (calculation details are listed in the ESI†). The *d-exps* are quite close to *d-cals* at lower concentrations of PW_12_ (20–30 wt%) while they deviate from each other at higher concentrations (40–70 wt%). The PW_12_ clusters are highly negatively charged and the electrostatic repulsion among PW_12_ clusters contributes to the deviations. The polarity of the PEG melt is much lower than that of water and therefore, the association of counterions (protons) to PW_12_ is stronger in PEG than in aqueous media, significantly shortening the effective distance of electrostatic interactions among PW_12_ clusters. Until the concentration of PW_12_ reaches ∼40 wt% in PEG, the clusters are close enough (1.82 nm for center-to-center distance; ∼0.8 nm for the closest surface-to-surface distance) to each other and inter-cluster repulsion can be observed. Moreover, the mass concentration of PW_12_ in the PEG melt is forced to be above 80% and the featured sharp diffraction peaks of PW_12_ crystals can be observed, suggesting the limit of solubility of PW_12_ in the PEG melt. The distance between the clusters is further reduced upon increasing the concentration to 80 wt%, and then PW_12_ will be inclined to aggregate and form insoluble crystals.

The high solubility of PW_12_ in the PEG melt originates from the multiple HB between the two components at the molecular scale. FT-IR measurements are carried out to probe the newly formed chemical bonding between PW_12_ and PEG (Fig. S1 in the ESI†). As the evidence of the stability of PEG400 in nanocomposites, the absorption band of the stretching vibration of C–O–C for both pure PEG400 and PEG400–PW_12_ nanocomposites appears at 1107 cm^–1^ (Fig. S1 in ESI†).^51^ In a typical IR spectrum of pure PW_12_, the W–O bonds of its three kinds of oxo ligands (O_t_, terminal oxo to W; O_c_, corner sharing oxo; O_e_, edge sharing oxo) give rise to characteristic vibrational bands at 978 cm^–1^, 896 cm^–1^ and 802 cm^–1^, respectively (Fig. S1 in the ESI†).^52^ The FT-IR spectra of PEG–PW_12_ nanocomposites show three similar characteristic bands of POMs, indicating the structural integrity of PW_12_ in the nanocomposites. Interestingly, the absorption peak at 802 cm^–1^, corresponding to the vibration of W–O_c_ in PW_12_, becomes sharper and shifts to 822 cm^–1^ (for PEG400–20% PW_12_), implying the HB interaction between PEG and O_c_ (Fig. S1 in the ESI†). As the bridging oxo ligand, O_c_ has a comparatively higher negative charge and thus behaves as an HB acceptor group.^53^ The enriched oxo ligands of PW_12_ can form HB with the ending hydroxyl groups of PEG, contributing to the high solubility of PW_12_ in the PEG melt. The sample can stay in a clear state without aggregation in a vacuum for at least half a year. The hydrogen bonding between PEG and POM and the electrostatic interaction among POMs can guarantee the stability of the nanocomposites.

### Proton conductive performances

**Fig. 2:**
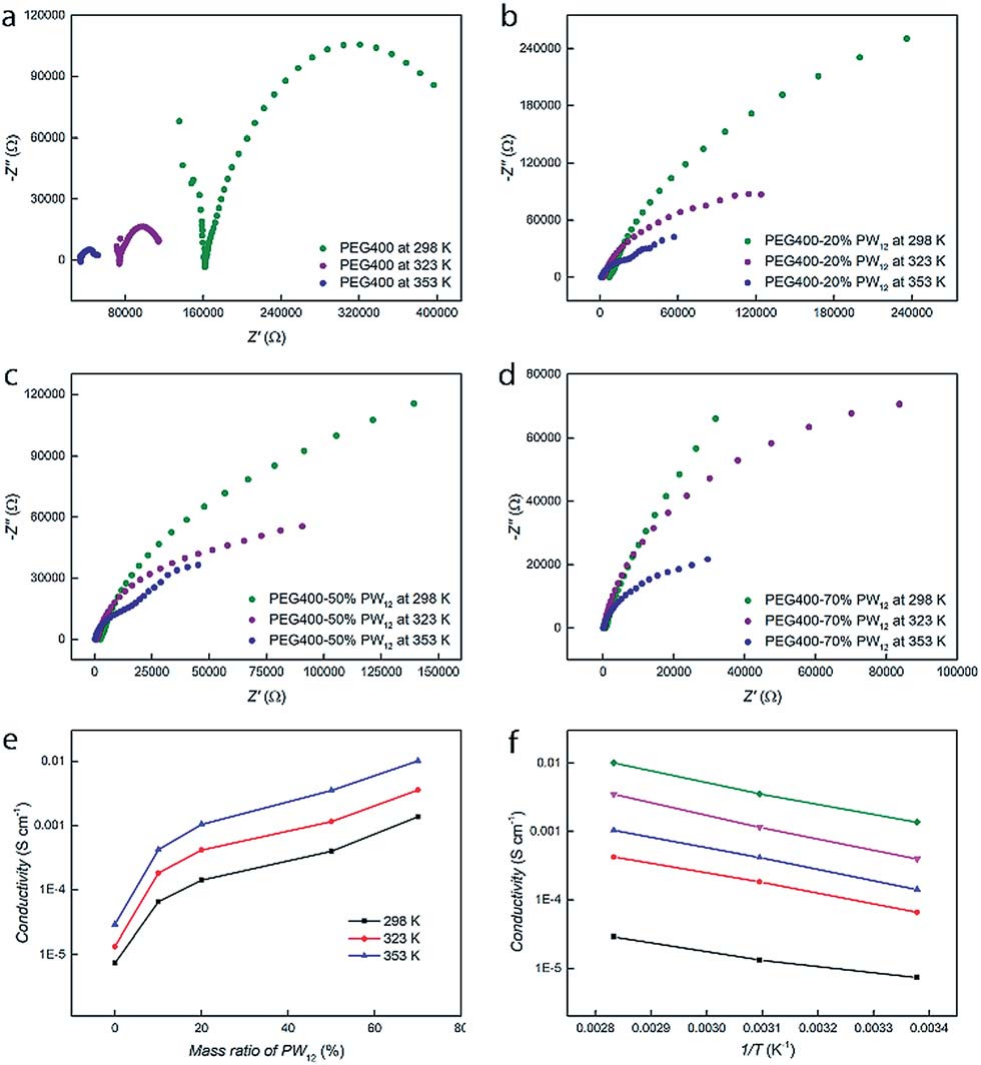
(a–d) Impedance spectra of PEG400–PW_12_ nanocomposites with different PW_12_ concentrations at 298 K, 323 K and 353 K, respectively. (e) The relationship between the conductivities and mass ratios of PW_12_ in the nanocomposites at different temperatures. (f) Arrhenius plots of conductivities for PEG400–PW_12_ with different PW_12_ concentrations (black for pure PEG400, red for PEG400–10% PW_12_, blue for PEG400–20% PW_12_, rose pink for PEG400–50% PW_12_, and green for PEG400–70% PW_12_).

Electrodes are immersed in the nanocomposites of PEG–PW_12_ and their proton conductivities are obtained *via* typical Nyquist plot analysis of the original data.^54^ The Nyquist plots of the impedance spectra of pure PEG and PEG400–PW_12_ composites with different PW_12_ concentrations (10%, 20%, and 70 wt%) at different temperatures (298 K, 323 K and 353 K) are summarized in Fig. 2a–d. Obviously, the doping of PW_12_ in the matrix of PEG can significantly enhance the conductivity of PEG from 7.6 × 10^–6^ (pure PEG) to 1.4 × 10^–3^ S cm^–1^ (70 wt% PW_12_) under ambient conditions (Fig. 2e). Interestingly, as the test temperature increased to the typical operation temperature of proton exchange membranes (353 K), the conductivity of the highly concentrated composite (70 wt%) is as high as 1.01 × 10^–2^ S cm^–1^, approaching the commercial requirements for proton conductive materials (Fig. 2e, Table S4 in the ESI†).^46^ The addition of PW_12_ not only increases the concentration of protons, but also provides scattered transporting sites that facilitate the mobilization of protons. The proton conductivities of PEG and nanocomposites show a dramatic increase from pure PEG to nanocomposites with low PW_12_ concentrations (10% and 20 wt%) while only a steady, comparatively tiny increase can be seen in the proton conductive performances of all the nanocomposites (from 10% to 70 wt%) (Fig. 2e). In the nanocomposites, PW_12_ clusters play the role of ‘supply stations’ and ‘transfer stations’ of protons, both contributing to the enhancement of the materials' conductivities. Meanwhile, PEG serves as the media for the dispersion of PW_12_, dominating the transport process of protons.^41^ In the nanocomposites of PEG400–PW_12_, the proton conduction performance is dependent on the proton concentration and proton transport process, which are further correlated to PW_12_ concentrations and the dynamics of PEG. When the concentrations of PW_12_ go higher in the nanocomposites, their proton conductivities are mainly limited by the proton transportation process in PEG media and therefore, we do not see huge increases as the concentrations of PW_12_ increase from 20% to 70 wt% (Fig. 2e).

To gain further mechanistic insight into the proton transportation process, thermodynamic studies on the nanocomposites have been carried out. Temperature-dependent proton conductivity measurements are carried out from 298 K to 353 K and it is clear that the conductivities increase exponentially with temperature. Typical Arrhenius plots of pure PEG and PEG–PW_12_ nanocomposites are shown in Fig. 2f. The activation energy (*E*_a_) of the proton conduction process, given by the slope of the plots, is similar for all the samples, including PEG400 and PEG–PW_12_ nanocomposites (0.094 eV for PEG400, 0.128 eV for PEG400–10% PW_12_, 0.137 eV for PEG400–20% PW_12_, 0.150 eV for PEG400–50% PW_12_, and 0.137 eV for PEG400–70% PW_12_), indicating that the dynamics of PEG chains contribute majorly to the proton conduction process of the hybrid nanocomposites. Meanwhile, the activation energy of the PEG@POM hybrid, which is constructed by encapsulating PEG molecules inside the nano-channels defined by POMs, is around 0.96 eV.^41^ This value is much higher than that of PEG400–PW_12_, implying that PEG chains show much faster dynamics in the newly developed system and facilitate rapid proton transportation.

There are two principal mechanisms to explain the proton transportation process: one case is the assistance of proton migration by translational dynamics of the host species, wherein the proton is transported in a vehicle, called the vehicle mechanism; the other one is the Grotthuss mechanism, in which case the protons diffuse through hydrogen bonds from one vehicle to the other and show local dynamics but reside on their sites.^2^,^4^ Our concept is different from these two mechanisms due to the existence of polymer chains. It was reported in our recent work that the localized longitudinal random walk of the chain segment of PEG, which exists as a distorted helix in the confining space of POMs, facilitates the proton conduction along the PEG backbones in the PEG@POM crystalline hybrids.^41^ Being like the Grotthuss mechanism, the proton conduction of POMs in PEG400–PW_12_ nanocomposites highly relies on the successful hopping of protons among POM clusters, which can form hydrogen bonds with PEG *via* their bridged oxo ligands. Meanwhile, protons can temporarily associate with an O atom on PEG and be transported along the polymer segments. The movement of PEG chain segments is also critical to the transportation of protons from one POM to its neighboring POMs, which is typical for the vehicle mechanism. Compared to those in PEG@POM crystalline materials, PEG chains in the newly developed PEG400–PW_12_ system are not confined and show much faster dynamics, facilitating rapid proton transportation. Therefore, the conductive performances of the newly obtained nanocomposites of PEG400–PW_12_ melt are better than those of the previous POM–PEG crystalline samples.

### Mechanical properties and processabilities

**Fig. 3:**
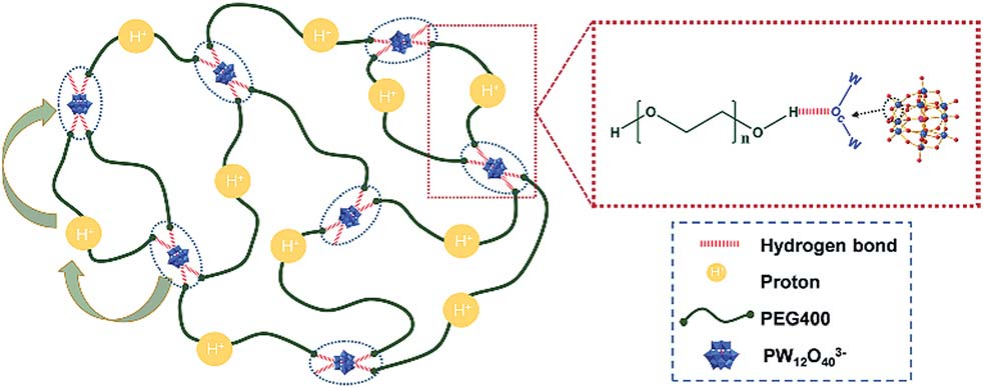
Schematic of the transfer of protons in PEG400–PW_12_ nanocomposites.

**Fig. 4:**
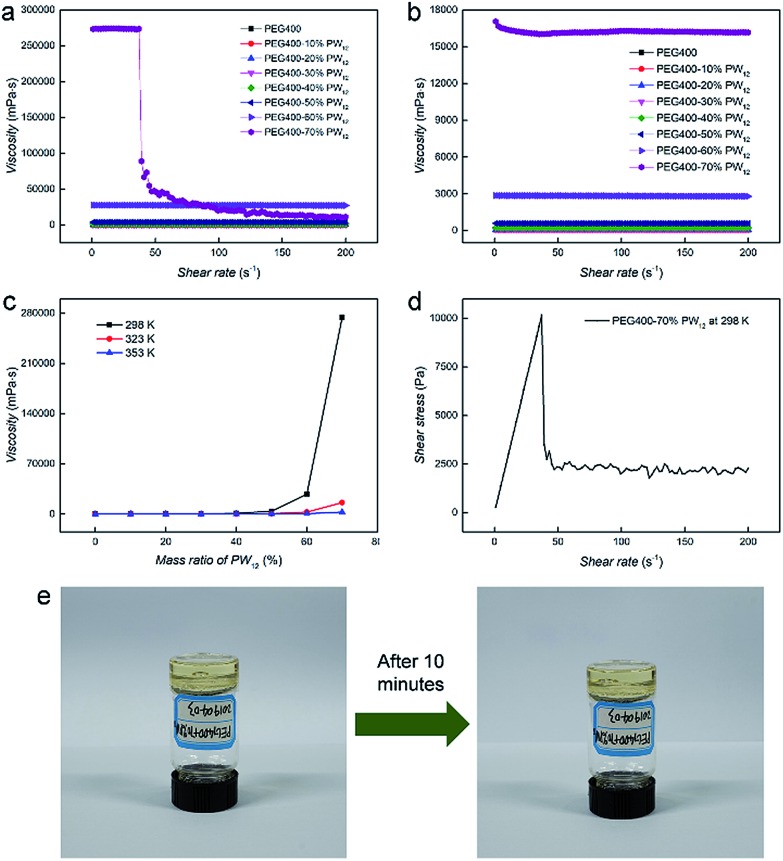
The flow curves of PEG400–PW_12_ nanocomposites at 298 K (a) and 353 K (b), respectively; (c) the relationship between viscosities and mass ratios of PW_12_ in the nanocomposites at different temperatures; (d) the relationship between the shear rate and shear stress for the PEG400–70% PW_12_ nanocomposite; (e) photographs of the PEG400–70% PW_12_ nanocomposite and the photographs show the highly viscous state of the sample.

The flowing properties under an external stimulus represent the key factors to evaluate the application and safety of SPCs in power devices as well as their processabilities. Therefore, rheometers are applied to examine the properties of nanocomposites and pure PEG under different shear rates and temperatures. Fig. 4a and b present the flow curves of PEG400–PW_12_ nanocomposites at 298 K and 353 K, respectively. The viscosity of the pure PEG400 sample is solely a function of temperature and is independent of the shear rate. The linear relationship between shear stress and shear rate indicates that PEG400 is a simple Newtonian fluid (Fig. S6 in the ESI†). For PEG400–PW_12_ nanocomposites, the shear viscosities of the nanocomposites are improved greatly with the increase of the mass ratio of PW_12_ (Fig. 4c). PEG400–PW_12_ nanocomposites with different PW_12_ concentrations (10% to 60 wt%) are still Newtonian fluids as seen from the linear relationship between shear stress and shear rate (Fig. S6 in the ESI†). However, PEG400–70% PW_12_ exhibits a remarkable shearing thinning behavior at 298 K (Fig. 4a and d). Specially, the shear viscosity remains almost unchanged at a lower shear rate, but decreases drastically over a shear viscosity of 32 s^–1^ which is typical of pseudoplastic fluids. The viscosity of PEG400–70% PW_12_ is 273 Pa s at 298 K which is high enough for the sample to behave like a solid at the static state and low shear state. As shown in Fig. 4e, PEG400–70% PW_12_ at the bottom of the container can be inverted and stay stationary for 10 minutes without an obvious flow, confirming that the SPC will not show flowability under a low flow (0–32 s^–1^). This property can be used to prevent the risk of leakage of PEG400–PW_12_ as proton conductors. As the concentration of PW_12_ increase to 70 wt%, the multiple HB between PEG and PW_12_ clusters can form dynamic crosslinked networks, which significantly strengthen the mechanical properties of the nanocomposites and thus prevent the orientation of PEG at a comparatively low shear rate (Fig. 3). However, at a high shear rate, the dynamic HB networks are deformed and re-arranged, significantly lowering the macroscopic viscosity of the nanocomposite (Fig. 4d, and Movie S1 in the ESI†). This typical flowing zone of the highly concentrated PEG–PW_12_ sample provides us the capability to easily process SPCs at a high shear rate. The viscosity of PEG–70% PW_12_ is *ca.* 10–100 Pa s at a higher shear rate, which is low enough for the composite to show flowability. The low viscosity and high flowability of the as-synthesized nanocomposite under high shear rate processing conditions facilitate the full contact between SPCs and electrodes, ensuring the high-standard performance of the resulting devices.

Generally, there are two factors that contribute to the increase of viscosity: one is the high-volume fraction of inorganic component-PW_12_ and the other is the HB interaction between PEG and POM.^55^ Similar to typical supramolecular interactions, HB is basically temperature sensitive and dynamic. Regarding the first feature, HB becomes weaker at higher temperature, losing its power to resist shear forces (Fig. 4b and S8 in the ESI†). Meanwhile, at high temperature (∼353 K), the relationship between the shear rate and stress of PEG400–PW_12_ (70 wt%) tends to be linear regardless of the slight fluctuation of data (Fig. S7 in ESI†), confirming the HB interaction between PW_12_ and PEG. As for the dynamic nature of HB, the restorability of HB networks in the nanocomposites is confirmed by measuring the shear stress while the shear rate circularly scans from 1–38 s^–1^ in typical rheological studies (Fig. S9 in the ESI†). The viscosity of PEG400–70% PW_12_ can basically remain invariable at 1–32 s^–1^ in the loop test, indicating that the HB networks can recovery rapidly when the shear rate decreases below the failure point (∼32 s^–1^). The self-restoring ability of HB networks is a key factor to ensure that the conductivities of nanocomposites are not affected by processing procedures.

### Structure–performance relationship at the molecular scale

Although the overall recipe for this hybrid system is simple and the synthesis is straightforward and convenient, the obtained materials are designed with high proton conductivities and tunable flowabilities, enabling cost-effective processing and stable device performances. The molecular features endow PW_12_ with strong acidic properties with their counterions (protons) loosely interacting with PW_12_ and thus the ‘free’ protons are quite dispersible in PEG media. These enriched protons finally contribute to high conductivities of these composites. Meanwhile, the mild electrostatic interaction between PW_12_ clusters and protons permits both the inward and outward diffusion of protons around the clusters, facilitating the transportation of protons in the nanocomposites.

Different from regular inorganic nanoparticle systems used in SPCs, PW_12_ clusters possess sub-nanometer scale sizes and enriched surface oxo ligands, enabling the formation of densely packed HB networks with PEG on their surfaces.^30^ This will finally promote the compatibility of PW_12_ to PEG media and the formation of HB networks, leading to the high stability of PEG–PW_12_ nanocomposites over long-term device operation and their dynamic rheological performances. Finally, it can be clearly seen that the sub-nm scale size of PW_12_ and its well-defined interaction with PEG are key to the smart performances of these novel SPCs. The SPCs here will majorly be used for electrolytes in batteries and super-capacitors. By strengthening the POM/PEG interaction, we could increase the elasticity of the nanocomposites, which can be also used as a proton exchange membrane in fuel cells.

## Conclusions

In summary, a simple inorganic cluster–PEG system provides a promising and convenient approach for developing SPCs with devisable mechanical and conductive performances. The conductivity of the nanocomposites can reach up to 1.01 × 10^–2^ S cm^–1^ with rapid proton transportation facilitated by the fast dynamics of polymer chains. Meanwhile, the dynamic nature of HB networks among inorganic and polymer components leads to the pseudo-plastic properties of the nanocomposites, which ensure the safety of devices by showing negligible flow at the static state or low shear rate state while the wettability to electrodes and cost-effective processing of the nanocomposites are expected from their high flowability (low viscosity) at high shear rates. The introduction of sub-nm clusters provides new opportunities for extending the versatility and controllability of SPCs.

## Conflicts of interest

There are no conflicts to declare.

## Supplementary Material

Supplementary information

Supplementary movie
