# Supplementary material for: Poly(ethylene glycol) nanocomposites of sub-nanometer metal oxide clusters for dynamic semi-solid proton conductive electrolytes
Source: Chem Sci. 2019 Jul 11;10(31):7333–9. doi: 10.1039/c9sc02779c (PMC6836960; doi:10.1039/c9sc02779c)
Supplement: Supplementary file 1 [file SC-010-C9SC02779C-s001.pdf]

Electronic Supplementary Material (ESI) for Chemical Science.
This journal is © The Royal Society of Chemistry 2019

Electronic Supplementary Information

Poly(Ethylene Glycol) Nanocomposites of Sub-Nanometer Metal Oxide Clusters for Dynamic Semi-Solid Proton Conductive Electrolytes

Zhao Zheng, [a] *Qianjie Zhou,* [a] *Mu Li* [a] *and Panchao Yin* *[a]

a. *South China Advanced Institute for Soft Matter Science and Technology & State Key Laboratory of Luminescent Materials and Devices, South China University of Technology, Guangzhou, 510640, P. R. China.*

Content

1. Details for MOV S1 2
2. Samples used in this work 2
3. The *d-cals* calculation method 2
4. The FT-IR spectra of PEG400, PW_{12} and PEG400-PW_{12} nanocomposites 4
5. Impedance spectra of PEG400-70%PW_{12} nanocomposites and the table of the conductivities of the PEG400-PW_{12} nanocomposites 4
6. DLS data of the PEG400-PW_{12} nanocomposites 5
7. The flow curves of PEG400-70%PW_{12} nanocomposites at 323 K 6
8. The relationship between shear rate and shear stress 6
9. The relationship between viscosities and volume fraction of PW_{12} in the nanocomposites 7
10. Cyclic curves of shear rate and shear stress 7
11. *Ea* of the PEG400-PW_{12} nanocomposites 8
12. Fitting curves of flow curves 9

1. Details for MOV S1

PEG400-70%PW_{12} nanocomposite behaves like solid with negligible flow at static state which is showed in Fig. 4e. Meanwhile, the shear thinning property of this sample enables its convenient processability and wettability to electrodes. There is a video MOV S1 in the attachments, which shows the fluidity of PEG400-70%PW_{12} composite under an external shear force in a syringe. These typical characteristics of pseudo-plastic fluid makes PEG400-PW_{12} an ideal candidate for semi-solid proton conductors.

2. Samples used in this work

Table S1. Samples used in this work.

Sample	PEG400 [g]	$H_3PW_{12}O_{40}$ [g]
PEG400	10	0
PEG400-10%PW_{12}	9	1
PEG400-20%PW_{12}	8	2
PEG400-30%PW_{12}	7	3
PEG400-40%PW_{12}	6	4
PEG400-50%PW_{12}	5	5
PEG400-60%PW_{12}	4	6
PEG400-70%PW_{12}	3	7
PEG400-80%PW_{12}	2	8

3. The *d-cals* calculation method

The density of PEG400: ρ (PEO) = 1.279 g cm^{-3}

The density of PW_{12}: ρ (PW_{12}) = 7.16 g cm^{-3}

Based on these values, the calculation method of the distance between PW_{12} is shown as bellow:

The number of PW_{12}:

$$Number\ of\ PW_{12} = \frac{Mass\ of\ PW_{12}}{Molecular\ weight\ of\ PW_{12}} \times N_A$$

The volume per PW_{12}:

$$Volume\ per\ PW_{12} = \frac{\frac{Mass\ of\ PEG400}{\rho(PEG400)} + \frac{Mass\ of\ PW_{12}}{\rho(PW_{12})}}{Number\ of\ PW_{12}}$$

The distance between PW_{12}:

$$d - cal = \sqrt[3]{Volume\ per\ PW_{12}}$$

The parameters were normalized with the value of *d-cal* of PEG400-20%PW_{12} as 1.

The *d-cals* of nanocomposites are listed in the Table S2 below:

Table S2. The *d-cals* of nanocomposites.

Sample	*d-cals*
PEG400-20%PW_{12}	1
PEG400-30%PW_{12}	0.8258
PEG400-40%PW_{12}	0.7381
PEG400-50%PW_{12}	0.6553
PEG400-60%PW_{12}	0.5865
PEG400-70%PW_{12}	0.5259

The *d-exps* of nanocomposites are listed in the Table S3 below:

Table S3. The *d-exps* of nanocomposites.

Sample	*d-exps*
PEG400-20%PW_{12}	1
PEG400-30%PW_{12}	0.8266
PEG400-40%PW_{12}	0.7646
PEG400-50%PW_{12}	0.6975
PEG400-60%PW_{12}	0.6245
PEG400-70%PW_{12}	0.5697

4. The FT-IR spectra of PEG400, PW_{12} and PEG400-PW_{12} nanocomposites

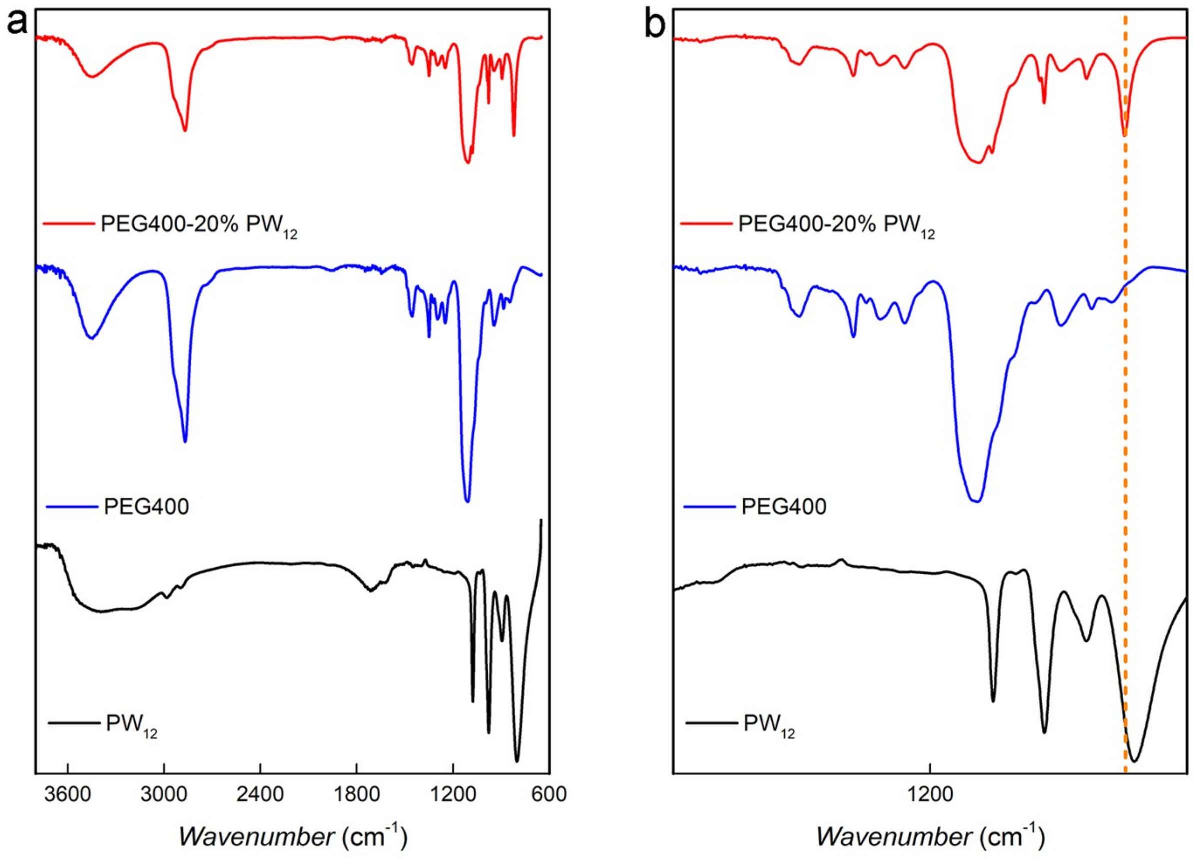

Fig. S1 FTIR spectra of PEG400, PW_{12} and PEG4000-20%PW_{12}.

5. Impedance spectra of PEG400-70%PW_{12} nanocomposites and the table of the conductivities of the PEG400-PW_{12} nanocomposites

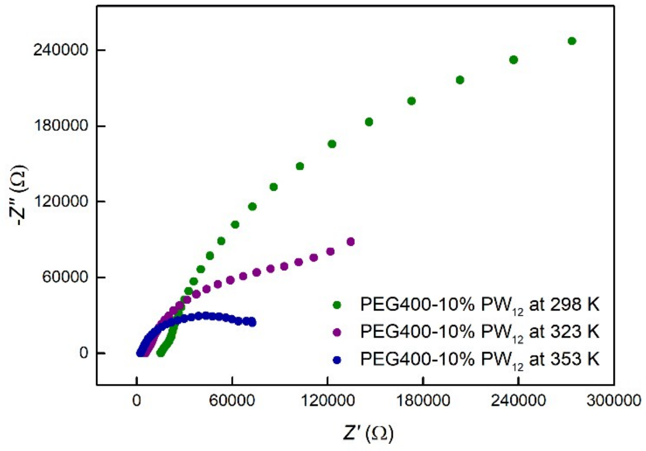

Fig. S2 Impedance spectra of PEG400-70%PW_{12} nanocomposites.

Table S4. The conductivities (σ) of the PEG400-PW_{12} nanocomposites.

Sample	σ at 298 K [S·cm^{-1}]	σ at 323 K [S·cm^{-1}]	σ at 353K [S·cm^{-1}]
PEG400	7.4×10^{-6}	1.3×10^{-5}	2.9×10^{-5}
PEG400-10%PW_{12}	6.5×10^{-5}	1.8×10^{-4}	4.2×10^{-4}
PEG400-20%PW_{12}	1.4×10^{-4}	4.2×10^{-4}	1.0×10^{-3}
PEG400-50%PW_{12}	4.0×10^{-4}	1.2×10^{-3}	3.5×10^{-3}
PEG400-70%PW_{12}	1.4×10^{-3}	3.6×10^{-3}	1.0×10^{-2}

6. DLS data of the PEG400-PW_{12} nanocomposites

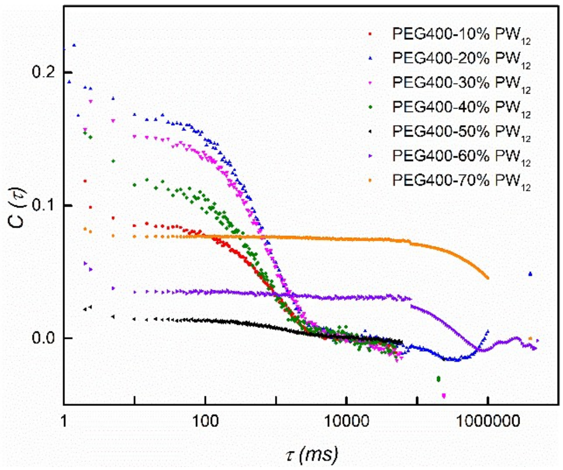

Fig. S3 The correlation functions of PEG400-PW_{12} nanocomposites (red for PEG400-10%PW_{12}, light blue for PEG400-20%PW_{12}, rose pink for PEG400-30%PW_{12}, green for PEG400-40%PW_{12}, black for PEG400-50%PW_{12}, purple for PEG400-60%PW_{12}, orange for PEG400-70%PW_{12}).

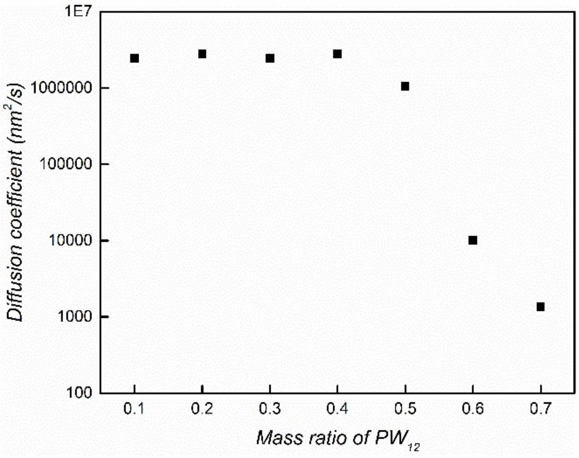

Fig. S4 The relationship between diffusion coefficients and mass ratio of PW_{12} in the nanocomposites.

7. The flow curves of PEG400-70%PW_{12} nanocomposites at 323 K

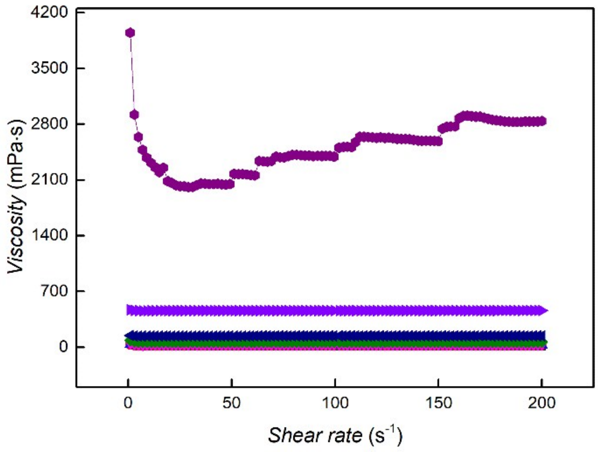

Fig. S5 The flow curves of PEG400-PW_{12} nanocomposites at 323 K (black for pure PEG400, red for PEG400-10%PW_{12}, light blue for PEG400-20%PW_{12}, rose pink for PEG400-30%PW_{12}, green for PEG400-40%PW_{12}, dark blue for PEG400-50%PW_{12}, light purple for PEG400-60%PW_{12}, dark purple for PEG400-70%PW_{12}).

8. The relationship between shear rate and shear stress

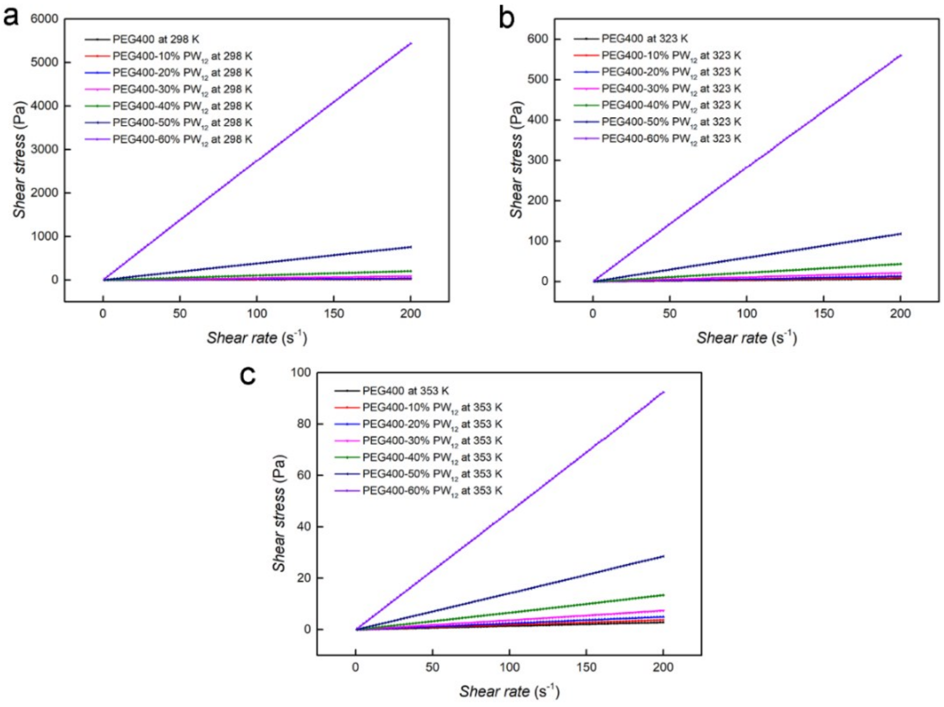

Fig. S6 The linear relationship between shear rate and shear stress for PEG400, PEG400-10%PW_{12}, PEG400-20%PW_{12}, PEG400-30%PW_{12}, PEG400-40%PW_{12}, PEG400-50%PW_{12}, and PEG400-60%PW_{12} at 298 K, 323 K and 353 K, respectively.

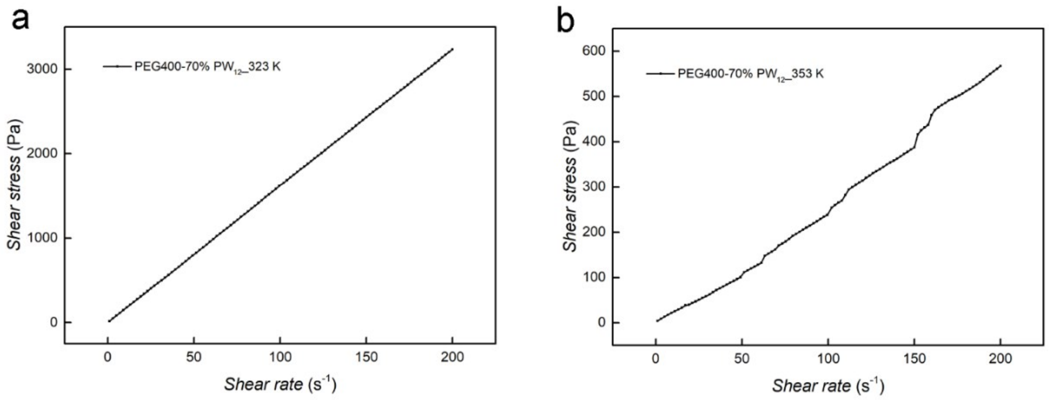

Fig. S7 The relationship between shear rate and shear stress for PEG400-70%PW_{12} at 323 K and 353 K, respectively.

9. The relationship between viscosities and volume fraction of PW_{12} in the nanocomposites

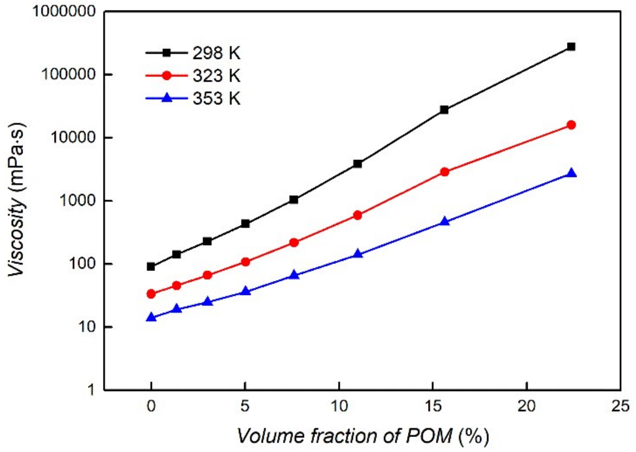

Fig. S8 The relationship between viscosities and volume fraction of PW_{12} in the nanocomposites at different temperature.

10. Cyclic curves of shear rate and shear stress

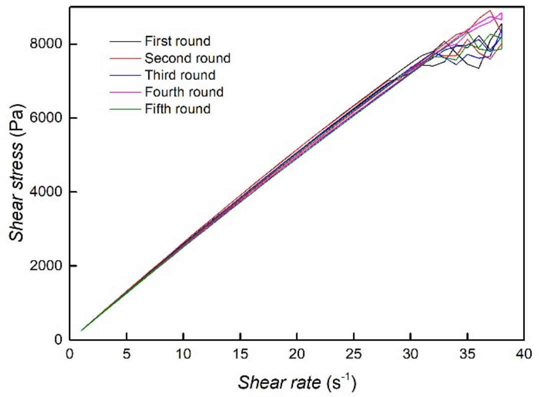

Fig. S9 The relationship between shear rate and shear stress for PEG400-70%PW_{12} nanocomposite.

11. *Ea* of the PEG400-PW_{12} nanocomposites

Table S5. *Ea* of PEG400-PW_{12} nanocomposites.

Sample	*Ea* [eV]
PEG400	0.094
PEG400-10%PW_{12}	0.128
PEG400-20%PW_{12}	0.137
PEG400-50%PW_{12}	0.150
PEG400-70%PW_{12}	0.137

12. Fitting curves of flow curves

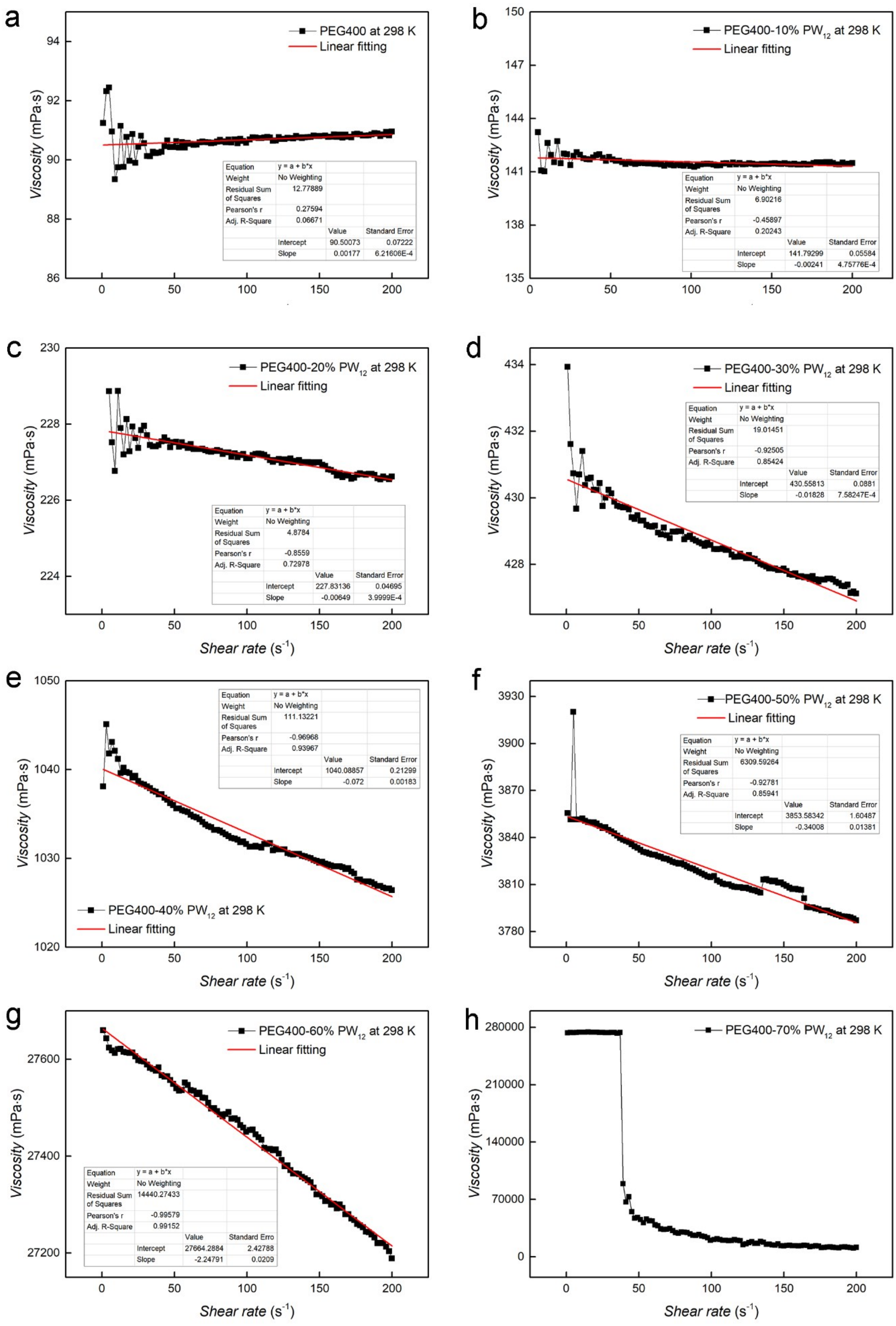

Fig. S10 Fitting of the flow curves of PEO400-PW_{12} nanocomposites at 298 K.

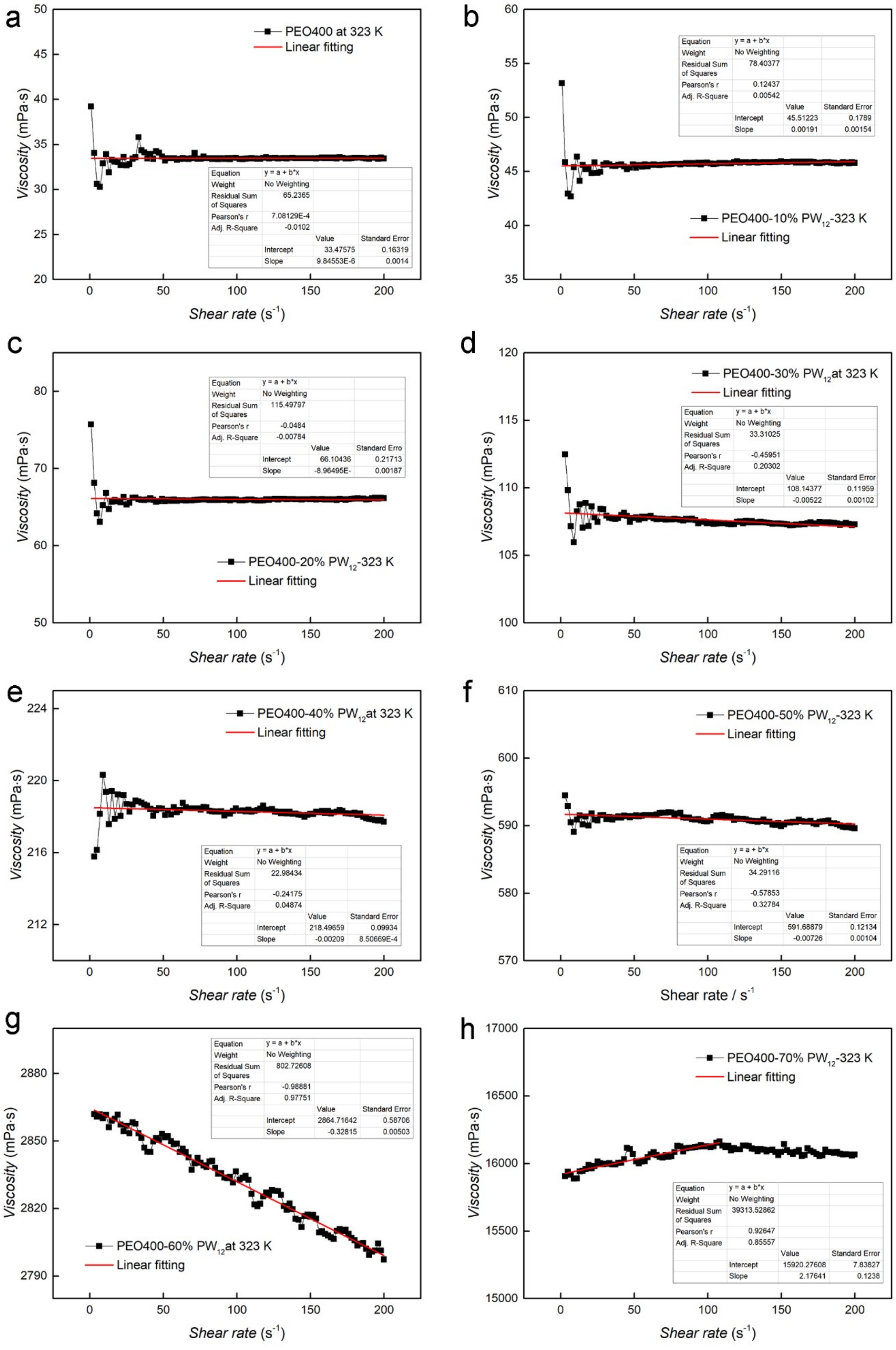

Fig. S11 Fitting of the flow curves of PEO400-PW_{12} nanocomposites at 323 K.

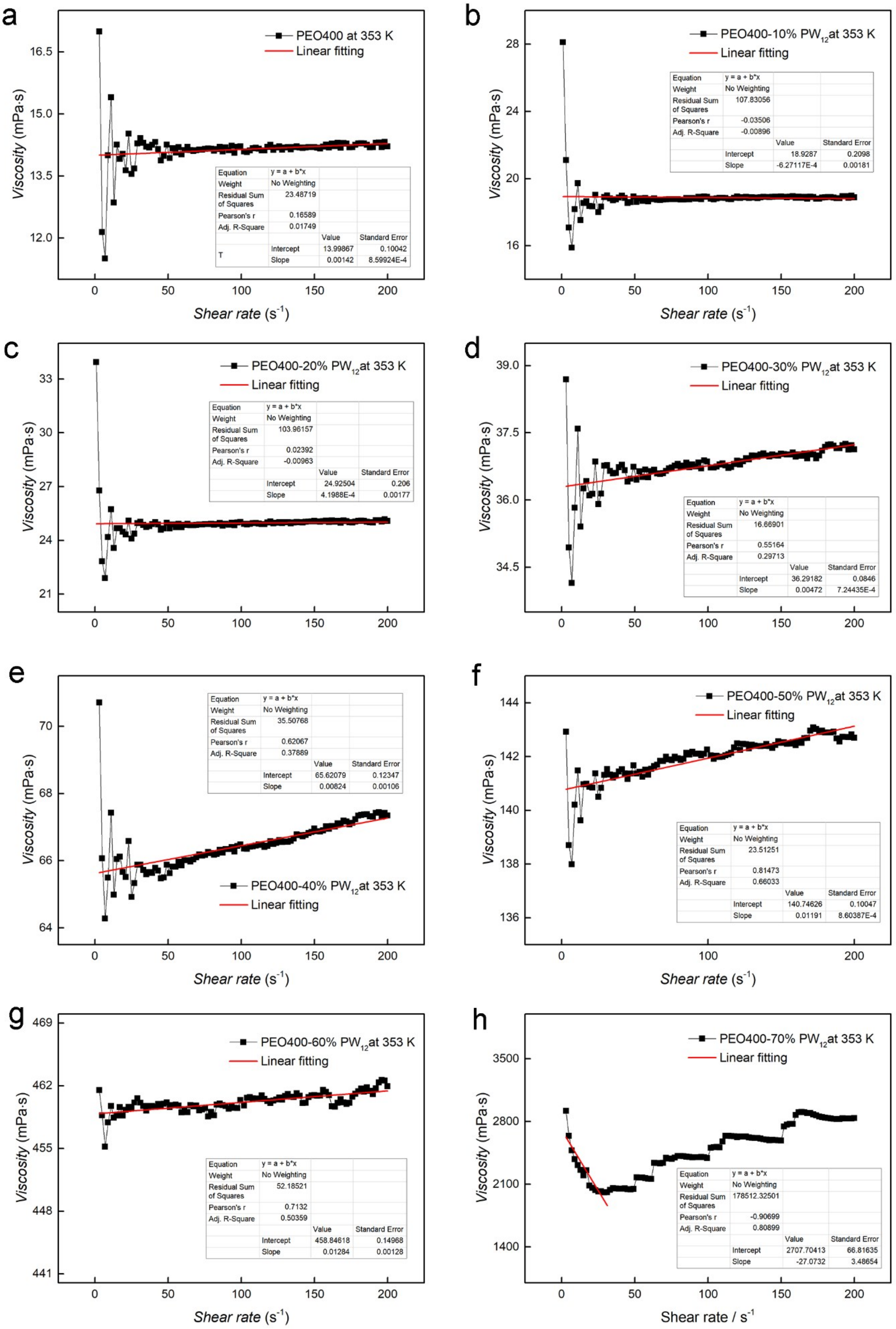

Fig. S12 Fitting of the flow curves of PEO400-PW_{12} nanocomposites at 353 K.
